# Current Molecular Targeted Therapy in Advanced Gastric Cancer: A Comprehensive Review of Therapeutic Mechanism, Clinical Trials, and Practical Application

**DOI:** 10.1155/2016/4105615

**Published:** 2016-01-10

**Authors:** Kaichun Li, Jin Li

**Affiliations:** ^1^Tianyou Hospital, Tongji University, Shanghai 200331, China; ^2^Fudan University Shanghai Cancer Center, Shanghai Medical College, Department of Medical Oncology, Shanghai 200032, China

## Abstract

Despite the great progress in the treatment of gastric cancer, it is still the third leading cause of cancer death worldwide. Patients often miss the opportunity for a surgical cure, because the cancer has already developed into advanced cancer when identified. Compared to best supportive care, chemotherapy can improve quality of life and prolong survival time, but the overall survival is often short. Due to the molecular study of gastric cancer, new molecular targeted drugs have entered the clinical use. Trastuzumab, an antibody targeting human epidermal growth factor receptor 2 (HER2), can significantly improve survival in advanced gastric cancer patients with HER2 overexpression. Second-line treatment of advanced gastric cancer with ramucirumab, an antibody targeting VEGFR-2, alone or in combination with paclitaxel, has been proved to provide a beneficial effect. The VEGFR-2 tyrosine kinase inhibitor, apatinib, can improve the survival of advanced gastric cancer patients after second-line chemotherapy failure. Unfortunately, none of the EGFR targeting antibodies (cetuximab or panitumumab), VEGF targeting monoclonal antibodies (bevacizumab), mTOR inhibitor (everolimus), or HGF/MET pathway targeting drugs has a significant survival benefit. Many other clinical trials based on molecular markers are underway. This review will summarize targeted therapies for advanced gastric cancer.

## 1. Introduction

Gastric cancer is the third leading cause of cancer death worldwide, followed by lung cancer and liver cancer. Gastric cancer is a common cancer, especially in East Asia, Middle and East Europe, and South America [1]. Annually, emerging cases of gastric cancer in China account for 44.2% of the global emerging gastric cases [2]. Although chemotherapy can improve survival in patients with advanced gastric cancer, the median overall survival is still less than 1 year [3]. Increasing numbers of studies have shown that molecular targeted therapy can further improve the survival of patients with gastric cancer. By summarizing data on HER2, VEGR, EGFR, mTOR, MET, and other common cancer targets or signaling pathways, this review attempts to expound the advance of molecular targeted therapy for gastric cancer in recent years.

## 2. Anti-HER2-Targeted Cancer Therapy

### 2.1. Trastuzumab (Herceptin)

Trastuzumab (Herceptin) is a recombinant humanized IgG1 monoclonal antibody directed against the extracellular domain of the human epidermal growth factor receptor type 2 (HER2; also known as ERBB2). Binding with high affinity to the extracellular domain of HER2, trastuzumab interrupts HER2-mediated cell signaling pathways and cell cycle progression, induces antibody-dependent cytotoxicity (ADCC), and then inhibits the proliferation of tumor cells that overexpress HER2 [4].

Trastuzumab was approved for the treatment of advanced gastric cancer by the US Food and Drug Administration (FDA) in November 2010 and by the Chinese version of the FDA in October 2012. Trastuzumab in combination with capecitabine or 5-fluorouracil and cisplatin is indicated for the treatment of patients with HER2-positive metastatic adenocarcinoma of the stomach or gastroesophageal junction. Trastuzumab is approved for use only in patients with metastatic gastric cancer with HER2 overexpression, which is defined as an immunohistochemistry (IHC) 3 positive result or an IHC 2 and fluorescence in situ hybridization (FISH) double-positive result.

ToGA was an open-label, international, phase III, randomized, controlled trial [5]. Patients with histologically confirmed, inoperable, locally advanced, recurrent, or metastatic adenocarcinoma of the stomach or gastroesophageal junction were selected. Patients were eligible if their Eastern Cooperative Oncology Group (ECOG) performance status was 0–2 and their tumor samples were HER2 IHC3 positive or FISH positive for HER2. In the 3883 screened patients, 810 were HER2-positive (22.1%). The 594 patients who satisfied all the eligibility criteria were randomly assigned in a 1 : 1 ratio to two groups: one group received chemotherapy alone, cisplatin (80 mg/m^2^) plus fluorouracil (800 mg/m^2^, Qd × 5 days), or capecitabine (1000 mg/m^2^, bid × 14 days); the other group received chemotherapy plus trastuzumab (Herceptin, F. Hoffmann-La Roche, Basel, Switzerland, 6 mg/kg, q3w). The primary endpoint was overall survival (OS), defined as time from randomization until death from any cause. Secondary endpoints included progression-free survival (PFS), time to progression, overall tumor response rate (RR), duration of response, and safety.

The results showed that the OS of the second group (which received trastuzumab plus chemotherapy) was 13.8 months, 2.7 months longer than that of the first group treated only with chemotherapy (calculated hazard ratio: HR = 0.74, *P* = 0.0046). There was an improvement in PFS from 5.5 months to 6.7 months by the addition of trastuzumab to chemotherapy (HR = 0.71, *P* = 0.002). A preplanned exploratory analysis according to HER2 level suggested that OS was longer in patients with high expression of HER2 protein compared to patients with low HER2 expression. The OS of the HER2 high-expression group (ICH2+/FISH+ or ICH3+) was 16.0 months. The adverse event profile was similar between the HER2 high-expression group and low-expression group. ToGA is a landmark study of gastric cancer. Its results demonstrate that trastuzumab plus chemotherapy can expand the overall survival of patients with advanced gastric cancer to more than 1 year and prove the important role of trastuzumab in gastric cancer therapy.

An additional ToGA study on Chinese [6] and Japanese [7] patients also showed that trastuzumab plus chemotherapy could improve the overall survival of patients with gastric cancer and the patients had a good drug tolerance.

Two phase II clinical trials were carried out to explore the efficacy and safety of trastuzumab plus XELOX (capecitabine/oxaliplatin) [8] and SP (S-1/cisplatin) [9] for advanced gastric cancer therapy, respectively. The efficacy, PFS, and OS of the combination groups were 67% and 68%, 9.8 and 7.8 months, and 21.0 and 16.0 months, respectively. The adverse event profiles were similar to ToGA. These results confirmed that trastuzumab plus XELOX or SP can also treat gastric cancer efficiently and safely. We expect further phase III clinical studies.

### 2.2. Lapatinib (Tykerb)

Lapatinib is an oral small molecule compound that inhibits epidermal growth factor receptor (EGFR) and HER2. By reducing tyrosine kinase phosphorylation of homodimers or heterodimers of the two receptors, lapatinib blocks EGFR or HER2 signal transduction, thereby inhibiting cell growth of gastric cancers that overexpress EGFR or HER2 and enhancing cancer cell apoptosis [10].

In 2013, at the ASCO annual meeting, researchers reported a phase III, randomized, double-blind trial evaluating the efficacy and safety of CapeOx in combination with lapatinib (CapeOx+L) or placebo (CapeOx+P) as a first-line treatment for advanced or metastatic HER2-positive gastric cancer patients (LOGiC Trial) [11]. The investigation showed that the median OS of patients treated with CapeOx+L and CapeOx+P was 12.2 and 10.5 months, respectively. Median PFS was 6.0 versus 5.4 months, respectively. ORR was 53% in the CapeOx+L arm and 40% in the CapeOx+P arm. Toxicity profiles were similar except for increased overall diarrhea and skin toxicity with CapeOx+L. These results indicate that lapatinib combined with CapeOx as first-line treatment had no survival benefit for HER2-positive advanced gastric cancer patients, though certain subgroups showed improvement.

TyTAN research is a phase III clinical study that explored the efficacy and safety of lapatinib in combination with chemotherapy to treat advanced HER2-positive gastric cancer as a second-line treatment [12]. Two hundred sixty-one HER2-positive patients were randomly assigned to receive paclitaxel (80 mg/m^2^) alone or in combination with lapatinib (1500 mg). The median OS, PFS, and time to progression (TTP) for these two groups were 11.0 versus 8.9 months, 5.4 versus 4.4 months, and 5.5 versus 4.4 months, respectively, without significant differences. The adverse event profile between these two groups was similar. These results suggested that lapatinib plus paclitaxel demonstrated activity in the second-line treatment of patients with HER2-positive advanced gastric cancer but did not significantly improve OS in the intent-to-treat population.

A phase II clinical trial in Germany showed that the efficacy of lapatinib plus capecitabine in patients with HER2-positive gastric cancer as second-line treatment was merely 11.1% [13]; in view of the depressing results, this study was winded up ahead of schedule. Neither LOGiC nor TyTAN research got positive results, probably because trastuzumab has cell-mediated antibody-dependent cytotoxicity (ADCC) while lapatinib lacks it.

## 3. Anti-VEGF Targeted Therapy

Abnormal angiogenesis is one of the basic characteristics of malignant tumors. Vascular endothelial growth factor (VEGF) secreted by tumor cells or tumor stromal cells is a key mediator of physiologic and pathologic angiogenesis [14].

There are two different clinical treatment strategies targeting VEGF: (1) using an anti-VEGF monoclonal antibody, such as bevacizumab, to inhibit the proangiogenic effect of VEGF; (2) using small tyrosine kinase inhibitor molecules (TKIs) (such as Apatinib) or VEGF receptor monoclonal antibodies (such as ramucirumab) to inhibit the function of the VEGF receptor. Another antiangiogenic drug is recombinant human endostatin, which was developed in China.

### 3.1. Avastin (Bevacizumab)

Professor Kang in South Korea described the Avastin in Gastric Cancer (AVAGAST) trial at the 2010 ASCO annual meeting. AVAGAST was a multinational, randomized, placebo-controlled phase III clinical trial designed to evaluate the efficacy of adding bevacizumab to capecitabine-cisplatin in the first-line treatment of advanced gastric cancer [15].

Seven hundred and seventy-four patients with advanced or metastatic adenocarcinoma of the stomach were enrolled in AVAGAST, and 387 were assigned to each treatment group. The two groups were treated with chemotherapy (capecitabine, 1000 mg/m^2^, bid, d1–14, q3w; plus cisplatin 80 mg/m^2^, d1, q3w × 6 cycle, and bevacizumab 7.5 mg/kg) or placebo. Efficacy measures showed improvements with bevacizumab and chemotherapy versus chemotherapy alone in the first-line treatment of advanced gastric cancer, including PFS (6.7 versus 5.3 months; HR, 0.80; *P* = 0.0037), and the response rate (46.0% versus 37.4%; *P* = 0.0315). However, they observed that adding bevacizumab to fluoropyrimidine-cisplatin only improved OS from 10.1 to 12.1 months in the placebo group (HR 0.87; 95% CI: 0.73–1.03; *P* = 0.1002), without reaching its primary objective. Therefore, bevacizumab plus chemotherapy could not significantly improve the overall survival of advanced gastric cancer patients.

Another phase III AVATAR study from China also found that bevacizumab combined with capecitabine/cisplatin chemotherapy did not significantly improve overall survival in patients with advanced gastric cancer [16].

One of the reasons for the failure of AVAGAST and AVATAR is that the two studies did not screen for important characteristics of the gastric cancer population. Biomarkers are urgently needed to select more suitable gastric cancer patients for bevacizumab treatment. We are looking forward to more in-depth basic research on molecular typing of gastric cancer cells. Based on studies such as AVAGAST, the US NCCN Gastric Guide (2015 Edition) does not recommend the use of bevacizumab in patients with advanced gastric cancer, although the FDA has approved bevacizumab for the treatment of advanced colorectal cancer and advanced lung cancer.

### 3.2. Apatinib

Apatinib is a small molecule tyrosine kinase inhibitor targeting VEGF-2, developed by Hengrui Pharmaceutical Company (China) [17]. Li et al. reported a phase II study that treated patients with metastatic adenocarcinoma gastric cancer with apatinib at the 2011 ASCO annual meeting [18]. One hundred and forty-four patients with advanced gastric cancer who failed at least two chemotherapeutic regimens were enrolled. The patients were randomized to receive placebo (group A) or 850 mg apatinib once daily (group B) or 425 mg apatinib twice daily (Group C).

In groups A, B, and C, the median OS was 2.50 months (95% CI, 1.87 to 3.70 months), 4.83 months (95% CI, 4.03 to 5.97 months), and 4.27 months (95% CI, 3.83 to 4.77 months), respectively, and the median PFS was 1.40 months (95% CI, 1.20 to 1.83 months), 3.67 months (95% CI, 2.17 to 6.80 months), and 3.20 months (95% CI, 2.37 to 4.53 months), respectively. There were statistically significant differences between the apatinib and placebo groups for both OS (*P* = 0.0017) and PFS (*P* < 0.001). Nine patients had a partial response (three patients in group B and six in group C). Toxicities were tolerable or could be clinically managed. The most common grades 3 to 4 adverse events were hand-foot syndrome and hypertension. Hematologic toxicities were moderate, and grades 3 to 4 hematologic toxicities were rare. These results demonstrated that apatinib improved PFS and OS in heavily pretreated patients with metastatic gastric cancer who had experienced treatment failure with two or more chemotherapy regimens.

At the 2014 ASCO annual meeting, Qin et al. reported the results of a phase III clinical study of treating metastatic adenocarcinoma gastric cancer with apatinib [19]. Two hundred and seventy-three patients with advanced gastric cancer who did not respond to second-line chemotherapy treatment were randomly assigned in a 2 : 1 ratio to receive oral apatinib (850 mg po qd, 28 days for one cycle) or placebo.

The trial showed that median OS was significantly longer by 55 days in the apatinib group compared with the placebo group (195 days versus 140 days; *P* < 0.016). Median PFS was also prolonged in the apatinib group compared with the placebo group (78 days versus 53 days; *P* < 0.0001). The objective response rates (ORR) of the apatinib group and the placebo group were 2.84% and 0.00%, respectively. The disease control rate was about 50%, although the efficiency was not so high. As for the safety, treatment with apatinib group was generally well tolerated. Most of the adverse events could be controlled by dose interruptions or reductions. Grades 3/4 adverse reactions that occurred in more than 2% of patients were hypertension, hand-foot syndrome, proteinuria, fatigue, anorexia, and elevated aminotransferase. Therefore, phase III study further confirmed the efficacy and safety of apatinib for patients with advanced gastric cancer.

In Asia, the number of patients with advanced gastric cancer who have experienced treatment failure with second-line chemotherapy is still rising. Therefore, effective follow-up treatments are urgently needed. Apatinib is the first-discovered small molecule inhibitor for VEGFR tyrosine kinase that has a clear effect for Asian patients with advanced gastric cancer. Although it prolonged OS by less than 2 months, this is remarkable progress for third-line treatment of advanced gastric cancer. We are expecting the efficacy and safety data of apatinib among Caucasians.

### 3.3. Ramucirumab (RAM, IMC-1121B, CYRAMZA)

VEGF and VEGF receptor-2 (VEGFR-2) mediates signal transduction and angiogenesis and can promote the development and progression of gastric cancer. Ramucirumab is a humanized monoclonal antibody that specifically blocks VEGFR-2 and downstream angiogenesis pathways [20].

Based on the REGARD study [21], in April 2014, the FDA approved ramucirumab (CYRAMZA, Eli Lilly) for the treatment of advanced gastric or gastroesophageal junction adenocarcinoma that developed after fluorouracil platinum therapy.

Then, in November 2014, based on the RAINBOW study [22], the FDA approved ramucirumab plus paclitaxel for the treatment of advanced stomach or gastroesophageal junction adenocarcinoma that developed after fluorouracil or platinum treatment. This is the only antiangiogenic drug that has been approved for advanced gastric cancer [23].

The REGARD study compared the efficacy and safety of ramucirumab versus placebo in the treatment of advanced gastric cancer (adenocarcinoma of the gastroesophageal junction) after a first-line chemotherapy failure. Three hundred and fifty-five patients who had received first-line chemotherapy, such as platinum or fluoropyrimidine drugs, but had cancer progression after chemotherapy, were randomly divided into a ramucirumab group (8 mg/kg, every 2 weeks by iv) and a placebo control group, and the primary outcome was overall survival. The results showed that the median OS in the ramucirumab group and the placebo group were 5.2 and 3.8 months (HR = 0.776, *P* = 0.047), respectively. Hypertension was more common in the ramucirumab group (16%), while hypertension in the control group affected only 8% (*n* = 9), and other adverse reactions were similar in the two groups.

For patients with advanced gastric cancer and esophageal and gastric adenocarcinoma, who failed first-line chemotherapy, ramucirumab is the first therapeutic monoclonal antibody used as a single agent to show a survival benefit. The research results confirm that VEGFR-2 is an important target for the treatment of advanced gastric cancer.

Another phase III RAINBOW study [22] found that the combination of ramucirumab with paclitaxel significantly improved the overall survival of patients with gastric cancer after failure of first-line chemotherapy treatment. Compared with treatment with paclitaxel, ramucirumab combined with paclitaxel can extend overall survival for 2.2 months (9.6 months versus 7.4 months, HR = 0.807, *P* = 0.017).

Treatment of advanced gastric cancer with ramucirumab alone or in combination with second-line paclitaxel has a certain curative effect [23]. Unfortunately, at the 2014 ASCO annual meeting, it was reported that ramucirumab combined with FOLFOX chemotherapy cannot improve the early cure median PFS in patients with advanced gastric cancer. A total of 168 patients with gastric or gastroesophageal junction adenocarcinoma without surgical resection of gastric or gastroesophageal junction were enrolled. For the patients treated with ramucirumab combined with FOLFOX and ramucirumab monotherapy, the median PFS was 6.4 months and 6.7 months, respectively (HR = 0.98, *P* = 0.89), and OS was 11.7 months and 11.5 months (HR = 1.08), respectively [24].

Interestingly, the AVAGAST study revealed that treatment of advanced gastric cancer with bevacizumab combined with first-line capecitabine/cisplatin showed no survival benefit, and similar results were obtained in advanced gastric cancer patients receiving first-line treatment with ramucirumab combined with the FOLFOX regimen. Molecular markers for screening for more suitable target patients are urgently needed for treatment with antiangiogenic agents.

### 3.4. Endostar (YH-16, Recombinant Human Endostatin)

Endostar is a novel recombinant human endostatin, which was approved for the treatment of non-small-cell lung cancer by China's Food and Drug Administration (CFDA) supervision bureau in 2005. Ling et al.'s study found that Endostar significantly inhibited VEGF-induced tumor cell proliferation and migration [25]. Furthermore, Endostar inhibited VEGF-induced KDR/Flk-1 (VEGFR-2) tyrosine phosphorylation and suppressed VEGFR-2 expression.

Xu et al. explored the efficacy and safety of Endostar combined with SOX (S-1/oxaliplatin) for the treatment of newly diagnosed patients with advanced gastric cancer [26]. A total of 165 patients with advanced gastric cancer randomly received chemotherapy treatment with Endostar combined with the SOX regimen or the SOX regimen alone. The primary study outcome was PFS. Their results showed that the PFS for the combination and monotherapy groups were 15 months and 12 months (*P* = 0.0001), respectively. However, the curative efficiency between the two groups was not significantly different (53.8% versus 42.4%, *P* = 0.188), with comparable adverse reactions. The conclusion is that the treatment of newly diagnosed advanced gastric cancer patients with Endostar combined with the SOX regimen is safe and effective. Few studies on the treatment of advanced gastric cancer with Endostatin combined with chemotherapy agents have been reported, and we are anticipating more studies focusing on the efficacy and safety evaluation of Endostatin.

### 3.5. Regorafenib (BAY 73-4506)

Regorafenib is an oral multikinase inhibitor with abroad catalytic action by activating series of protein kinases, such as VEGFR and RAF [27], with wide-spectrum anticancer activity in early-phase clinical trials. Expression of phosphorylated and total VEGFR-2 proteins was found significantly decreased in regorafenib treated mouse xenografts models, which were constructed by gastric cancer cells, so antiangiogenesis maybe the major driving force of regorafenib when performing antitumor function in gastric cancer [28].

To test the application value of regorafenib in treatment of refractory advanced oesophagogastric cancer, a randomized, phase II, double-blind, placebo-controlled study was carried out. The proportion of advanced gastric cancer in all enrolled patients was 85/147; at the endpoint, regorafenib group got significantly longer median PFS than placebo group (11 weeks versus 3.9 weeks, *P* < 0.0001) and the median OS (25 weeks versus 19.4 weeks, *P* = 0.11) also showed a positive trend [29]. However, for the whole enrolled patients, higher concentration of plasma VEGF-A did not make definite difference in treatment outcomes compared to the lower ones.

From the current research results, the prospect of regorafenib application in the treatment of intractable patients who experienced primary chemotherapy failure is good. But the drug-related sever adverse events are big problem which may discount patients' quality of life. It is urged for us to study another hard issues: what is the keenly functional molecular targets answer to regorafenib in advanced gastric cancer patients? And how to use it accurately? More clinical trials and usage improvement are needed for underlining the drug's value in the future.

## 4. Anti-EGFR-Targeted Therapy

The epidermal factor receptor (growth) family consists of four members, including EGFR (HER1), HER3, HER2, and HER4. Overexpression of EGFR often indicates a poor prognosis, which provides potential therapeutic targets for diseases [30]. Unfortunately, neither the anti-EGFR monoclonal antibodies (cetuximab and panitumumab) nor the inhibitor (lapatinib) antagonizing the downstream EGFR tyrosine kinase significantly improved the survival of patients with advanced gastric cancer.

### 4.1. Cetuximab (C-225, Erbitux)

Cetuximab is a recombinant human-mouse chimeric epidermal growth factor receptor (EGFR) IgG1 monoclonal antibody. EXPAND study explored the efficacy and safety of treatment of advanced gastric and gastroesophageal junction adenocarcinoma with cetuximab combined with XP regimens (gemcitabine/cisplatin) [31]. A total of 904 patients with locally advanced or metastatic gastric cancer or gastroesophageal junction carcinoma were enrolled. These patients were randomly divided into two groups receiving treatment with cetuximab combined with XP regimens or XP regimens alone. PFS was the primary outcome for this study. The results showed that PFS was lower in the XP group than that in the XP group (4.4 months versus 5.6 months, HR = 1.09, 95% CI 0.92–1.29, *P* = 0.32). the conclusion is that the treatment of advanced gastric cancer with the combination of cetuximab and XP regimens does not have a survival benefit.

### 4.2. Panitumumab (Vectibix, Amgen)

Panitumumab is a fully humanized epidermal growth factor receptor (EGFR) IgG2 monoclonal antibody. A REAL3 Research tested the efficacy and safety of treatment of advanced gastric and gastroesophageal junction adenocarcinoma with panitumumab combined with a united EOC scheme (epirubicin/oxaliplatin/card capecitabine) [32]. The results showed that the median OS in the combined treatment group was reduced by 2.5 months, compared with the EOC monotherapy group (8.8 months versus 11.3 months, HR = 1.37, 95% CI 1.07–1.76, *P* = 0.013).

EGFR is expressed in epithelial cells of gastric cancer at a relatively high level, and the mutation rate of KRAS in gastric cancer is much lower than that in colorectal cancer (4.2%) [33], and therefore EGFR-targeted therapy is the present research hot spot. Judging from the current studies, treatment of metastatic gastric cancer with either small molecule TKI or monoclonal antibodies showed no obvious curative effect. The main reason for the failure of REAL3 and EXPAND research might be that suitable targeted populations were not screened with appropriate biomarkers and that both studies have not evaluated patients for EGFR expression by IHC or FISH to further select the treatment group.

### 4.3. Nimotuzumab (h-R3)

On EGFR overexpressed tumor cells experiments in vitro, nimotuzumab showed remarkable cancer reversing effects such as antiproliferation, antiangiogenesis and promotes apoptosis [34]. Same as cetuximab, nimotuzumab is another kind of recombinant humanized monoclonal immunoglobulin G1 antibody, with high binding specificity to the human extracellular EGFR, displaying high killing effect in the EGFR positive cancer cells. But nimotuzumab displays a longer half-life, higher dose-effect rate, and less severe dermatological toxicity than cetuximab [35]. Several clinical trials have been performed to find the therapeutic benefit of nimotuzumab in patients with advanced gastric cancer. But the results vary with each chemotherapeutics. In 2014, in double-blind phase II trial [36], 83 irinotecan-naive patients with advanced gastric included 40 who received nimotuzumab 400 mg/week plus irinotecan 150 mg/m^2^/biweekly, and the rest of patients were treated by irinotecan 150 mg/m^2^/biweekly only. The results reflected that no difference in PFS and OS between these two randomly groups, but no more side effects were reported in nimotuzumab group. On the other hand, significant efficacy was detected in EGFR 2+/3+ subgroups compared to EGFR 0/1+ patients (median PFS: 118.5/59.0, OS: 358.5/229.5 days, resp.). A phase III trial of nimotuzumab and irinotecan as a second line treatment with advanced cancer have had been approved (ClinicalTrials.gov Identifier: NCT01813253), aiming to obtain further clinical evidences. As a conclusion so far, curative effect of nimotuzumab is proportional to the tumor surface EGFR density of patients, and the low adverse events rate allows patients who received nimotuzumab plus irinotecan to gain a well quality of life.

Recently, another randomized phase II trials performed to test if nimotuzumab can make a difference in CS (Cisplatin and S-1) treated patients with unresectable and metastatic gastric cancer [37]. The results showed that neither NCS (nimotuzumab plus CS) group nor EGFR 2+/3+ subgroup patients got additional benefit from nimotuzumab. This finding also raises a hypothesis that a negative interaction existed between nimotuzumab and S-1, so before getting more definite proof, anti-EGFR antibody drugs would not be commended to go with S-1 based chemotherapy.

## 5. Anti-mTOR-Targeted Therapy

It has been reported that the mammalian target of the rapamycin (mTOR) signaling pathway is frequently activated in gastric cancer cells. Preclinical research showed that, in in vitro studies and in animal models, blockage of the mTOR signaling pathway can inhibit the proliferation and metastasis of gastric cancer cells [38].

Everolimus (RAD001) is an oral mTOR inhibitor. A multicenter clinical phase II study explored the efficacy and safety of everolimus in the treatment of advanced gastric cancer [39]. Fifty-three advanced gastric cancer patients with previous chemotherapy failure were treated with everolimus (10 mg, oral), once a day, until disease progression. Despite no CR and PR cases, the disease control rate (DCR) was 56%, the median PFS was 2.7 months, and the median OS was 10.1 months. The treated patients usually had grades 3-4 adverse reactions, including anemia and hyponatremia and increase of the serum level of alanine aminotransferase and decrease in lymphocytes. The conclusion is that everolimus monotherapy has a good disease control rate for advanced gastric cancer patients with the failure of previous chemotherapy.

Unfortunately, an international multicenter, double-blind, randomized phase III GRANITE-1 study found that everolimus did not improve the survival of advanced gastric cancer patients who had failed previous chemotherapy [40]. Six hundred and fifty-six cases with failed first- or second-line chemotherapy were randomized to receive everolimus (10 mg/day) or placebo treatment. The main outcome was overall survival. Their results demonstrated that the OS was 5.4 months for the everolimus group and 4.3 months for the placebo group (HR = 0.90, *P* = 0.124). Median PFS was 1.7 months and 1.4 months (HR = 0.66) for the everolimus and the placebo group, respectively. Grades 3-4 adverse reactions including anemia, loss of appetite, and fatigue were observed in treated patients.

Although the FDA has approved everolimus for the treatment of advanced renal cell carcinomas that have not responded to treatment with other drugs (sorafenib and sunitinib) (March, 2009) [41] and hormone receptor positive and HER2-negative advanced postmenopausal breast cancer patients (July, 2012) [42] for the use of everolimus in the treatment of advanced gastric cancer; there is a need to select more suitable target populations.

## 6. Anti-HGF/MET-Targeted Therapy

The mesenchymal-epithelial transition factor receptor (MET) is activated by hepatocyte growth factor (HGF) and multiple signal pathways are activated upon HGF induction to promote gastric cancer cell proliferation, survival, and migration [43]. MET gene mutation, amplification, or overexpression can activate the deregulated MET pathway. The researchers have developed various kinds of inhibitors antagonizing HGF/MET pathways, including monoclonal antibodies (rilotumumab and onartuzumab) and small molecule tyrosine kinase inhibitors (foretinib).

### 6.1. Rilotumumab

Rilotumumab is a humanized monoclonal antibody directed against HGF. A phase II study assessed the efficacy and safety of rilotumumab combined with ECX scheme (epirubicin/cisplatin/capecitabine) in the treatment of advanced gastric cancer (adenocarcinoma of the gastroesophageal junction) [44]. A total of 121 patients were randomly assigned to receive a combination of high-dose rilotumumab (15 mg/kg) and ECX, low-dose rilotumumab (7.5 mg/kg) and ECX, or placebo and the ECX regimen. The results showed that the PFS was 5.1 months for a high dose of rilotumumab (HR = 0.69, *P* = 0.164) and 6.8 months for a low dose of rilotumumab (HR = 0.53, *P* = 0.009), compared with 4.2 months for the placebo group. Adverse events of grades 3-4 were more common in the rilotumumab group, including a reduction of neutrophils (44% versus 28%) and venous thromboembolism (20% versus 10%). The conclusion is that there are no extra adverse events and more antitumor activity in the treatment group with low-dose rilotumumab combined with ECX regimens.

At the 2015 ASCO annual meeting, Professor D. Cunningham introduced a global multicenter randomized phase III RILOMET-1 study [45]. A total of 609 patients with HER2-negative and MET-positive advanced gastric or gastroesophageal junction adenocarcinomas were randomly divided into two experimental groups, receiving rilotumumab plus ECX (epirubicin/cisplatin/capecitabine), or a control group (receiving only ECX) treatment. The main outcome was overall survival. It was found that the OS was not prolonged in the experimental group (9.6 versus 11.5 months, HR = 1.37, *P* = 0.021) compared with the placebo group. Moreover, the adverse reaction rate was much higher in the experimental group than in the control group. This study was terminated early in November 2014 because of deaths in the test group.

To explain the opposite results reported by phase III RILOMET-1 study and phase II study, one possible cause is that the IHC experiments performed in these two studies used different antibodies to detect MET expression in the tumor tissues. MET positive was defined as a >50% 1+ mount of cytoplasmic staining in phase II study, and 42% of the subjects were MET positive; however, MET positive was defined as a >25% 1+ mount of cell membrane staining in phase III study, and 71% of the subjects were MET positive. The 29% addition of “MET positive” patients in phase III study versus phase II study may dilute the survival benefit for real MET positive patients.

### 6.2. Onartuzumab

At the 2015 ASCO annual meeting, a poster communicated information about another global multicenter phase III MET Gastric study using anti-MET monoclonal antibody onartuzumab [46]. In this study, the efficacy and safety of treatment of metastatic HER2^−^MET^+^ advanced gastric cancer (adenocarcinoma of the gastroesophageal junction) with first-line mFOLFOX6 combined with onartuzumab were studied. It was found that the combination treatment with mFOLFOX6 and onartuzumab could not prolong the OS (11.0 versus 11.3 months, HR = 0.82, *P* = 0.244). Even in the MET highly expressed group (MET2+/3+), a survival benefit was not observed (*P* = 0.062). Adverse 3-4 level reactions, such as the reduction of neutrophils, thrombocytopenia, edema, and pulmonary embolism, were not significantly increased in the onartuzumab group.

The results from two phase III RILOMET-1 and MET Gastric studies were negative, and the development of HGF/MET-targeted monoclonal antibodies for the first-line treatment of advanced gastric cancer has been nearly stopped. The possible reasons for obtaining negative results in these two studies might be that the MET gene is not the driver gene for gastric cancer; HGF/MET signaling pathway cross-talks with other signaling pathways; there is no accurate enough prediction of MET protein expression; there is gastric cancer heterogeneity, and so on.

### 6.3. Foretinib

Foretinib (GSK1363089) is an oral multikinase inhibitor, which can inhibit multiple targets, including MET, VEGFR-2, RON, and AXL. Preclinical studies showed that foretinib could effectively inhibit the growth of gastric cancer cells by blocking the signal transduction pathway of tyrosine kinase [47]. Phase II clinical study found that the cMET/VEGFR-2 inhibitor foretinib could not improve the survival of patients with advanced gastric cancer without previous chemotherapy [48]. The exact effect of foretinib needs to be confirmed by further study.

## 7. Conclusion

Along with the development of gastric cancer research, more and more targeted drugs are being developed to target genes and signaling pathways. Over the past few years, clinical trials of multiple targeted agents have been completed or are being carried out for gastric cancer therapy, and these results have gradually changed the clinical treatment of gastric cancer. The Gastric Cancer Guide (2015 Edition) from the United States National Comprehensive Cancer Network (NCCN) recommended trastuzumab (first line) and ramucirumab (second line) for the treatment of advanced gastric cancer [49].

Trastuzumab improves the survival of patients with HER2-positive advanced gastric cancer, and treatment with trastuzumab is now considered to be the standard first-line therapy. However, two large studies found that the small molecule inhibitor lapatinib, also targeting HER2, did not reproduce the positive result found in the ToGA study. Positive results were not obtained from EGFR-, VEGF-A-, or mTOR-targeted drugs in early clinical trials. Fortunately, the anti-VEGFR-2 ramucirumab and the VEGFR-2 small molecule inhibitor apatinib bring hope to the treatment of advanced gastric cancer. Two negative phase III studies have recently been reported, which cast a shadow on the HGF/cMET pathway-targeted therapy for gastric cancer.

We look forward to discovering more gastric cancer driver genes and to designing corresponding targeted drugs for future basic research. We also anticipate greater progress of gastric cancer research in selected appropriate patients with targeted therapy.
